# The Role of Age and Social Motivation in Developmental Transitions in Young and Old Adulthood

**DOI:** 10.3389/fpsyg.2012.00366

**Published:** 2012-09-27

**Authors:** Jana Nikitin, Lea C. Burgermeister, Alexandra M. Freund

**Affiliations:** ^1^Institute of Psychology, University of ZurichZurich, Switzerland

**Keywords:** adult development, developmental transitions, social motivation, diary studies, social relationships

## Abstract

Two diary studies investigated the role of social approach and avoidance motivation in important developmental transitions in young and old adulthood. Study 1 comprised a sample of young adults (*N* = 93, *M* = 21.5 years) who moved out of their parental homes. The sample of Study 2 consisted of older adults (*N* = 69, *M* = 76.95 years) who moved into senior housing. In both studies, participants reported their habitual social approach and avoidance motives as well as their daily social experience and subjective well-being over the course of 2 weeks. In line with the literature, social approach motives and age were related to higher subjective well-being, whereas social avoidance motives were negatively associated with subjective well-being. Time since the transition was an important moderator of the association between social avoidance motives and negative outcomes. With increasing time from the transition, the negative effects of social avoidance motives decreased. The positive effects of social approach motives remained fairly stable over time. Importantly, age did not moderate any of the associations between social motivation and outcomes. Results are discussed in terms of transition-related instability and age-related stability.

## Introduction

Transitions – defined as going from a predictable (familiar) to an unpredictable (unfamiliar) context (Caspi and Moffitt, 1993) – involve experiences of novelty, ambiguity, and insecurity. New roles have to be adopted, unfamiliar situations to be mastered, new social relationships to be established, and new behaviors to be acquired and displayed. Successfully mastering a transition is dependent on many factors, ranging from intelligence (Pargas et al., 2010) to macro-economic circumstances (Schoon and Duckworth, 2010). Dispositional variables might also play an important role because they can help to establish stability in the new context by transforming new situations into familiar ones (Caspi and Moffitt, 1993).

We posit that social approach and avoidance motives – characterized as the dispositional hope for affiliation and the dispositional fear of rejection, respectively (Sokolowski et al., 2000) – are of central importance for understanding success or failure in transitions (for a more detailed discussion of the role of social motives for transitions see Nikitin and Freund, 2008). In short, this should be the case because social approach and avoidance motives affect the ability to establish and maintain interpersonal relationships (for a summary see Gable and Berkman, 2008), an aspect that is particularly important for adapting to transitions (Cutrona, 1982; Mitchell and Kemp, 2000) and for subjective well-being and physical health in general (House et al., 1988; Seeman and Crimmins, 2001; Cacioppo et al., 2002; Uchino, 2009). The current studies test if social approach motives have a positive impact on mastering a transition, whereas social avoidance motives exert a negative influence on adapting to a transition.

Although there is some evidence for the role of social approach and avoidance motives for transitions in young adulthood (Cutrona, 1982; Asendorpf and Wilpers, 1998), less is known about the role of social approach and avoidance motives for transitions in older adulthood or about possible age-related differences. There are three plausible alternative hypotheses regarding age-related differences in the impact of dispositions on mastering transitions. First, based on the assumption that, with increasing age, adults are more motivated for and competent in emotion regulation (Gross et al., 1997), the effects of dispositions on subjective well-being might become weaker across adulthood because the effects of emotion regulation might overwrite those of dispositions. In contrast, effects of dispositions might become stronger over time due to possible cumulative processes (Impett et al., 2010). Finally, the effects of dispositions might be stable over the life span (Nikitin and Freund, 2011). This hypothesis is based on Neugarten’s (1964) notion of the institutionalization of personality, assuming that personality traits and their interaction with the social environment stabilize with age. The first goal of the present paper is to test these three hypotheses against each other.

The present paper also addresses the question during which phase of the transition dispositions take a “center stage in the drama of life” (Caspi and Moffitt, 1993, p. 247). Following Caspi and Moffitt, we assume that the answer is paradoxical: dispositional continuity is most likely to emerge during periods of discontinuity in the social context. During transitions into new situations – when one has to behave in some way but no information is acquired yet about how to behave adequately – individual dispositions are most likely to be accentuated because they provide guidelines for how to read and interpret as well as act in novel, ambiguous, and uncertain situations (Caspi and Moffitt, 1993). According to this perspective, the impact of dispositions should wear off over time when more knowledge is acquired. To our knowledge, this hypothesis has not yet been investigated empirically. Therefore, the second goal of this paper is to test if the effects of dispositions on cognition and behavior decrease over the time of a transition.

## The Role of Social Approach and Avoidance Motives in Transitions

The ability to establish and maintain interpersonal relationships is one of the central milestones of successful development across the life span (Lang, 2004; Tesch-Römer, 2010). Building positive social relations might be particularly important in the transition from adolescence into young adulthood, as some of the central developmental tasks during this transition have a strong social component: establishing autonomy and independence from parents, building meaningful social ties and friendships with peers, establishing a romantic relationship, and being able to navigate social relations when starting studies at a university (Eccles et al., 2003). Establishing and maintaining social relationships is not only important during transitions in young but also in older adulthood, be it retirement (Wang et al., 2011), widowhood (Ha and Ingersoll-Dayton, 2011), or moving to senior housing or an assisted living facility (Mitchell and Kemp, 2000). Previous research found that subjective well-being and satisfaction with the new home depends on the quality of social relationships in the new environment (Mitchell and Kemp, 2000; Street et al., 2007). Regardless of age, then, it seems that if younger or older adults do not master these challenges during the transition, this results in feelings of loneliness and isolation (Cutrona, 1982; Larose and Boivin, 1998; Sergeant and Ekerdt, 2008; Burge and Street, 2010).

In order to investigate the role of social motives for adapting to central developmental transitions across adulthood, we compare young adults who move out of the parental home and older adults who move into senior housing facilities. These two transitions share certain aspects (i.e., moving into a new physical and social environment) but they also differ in a number of regards. For instance, the reasons for the move are different for the two age groups. Young adults who start university often move into a shared apartment because the university is far away from their parents’ home (Larose and Boivin, 1998). The reason for moving into senior housing in older adulthood is mostly a significant decrease in physical health and mobility (Pitts et al., 2005; Nygren and Iwarsson, 2009). However, both young and older adults who move have to face similar social challenges. Oftentimes, it is more difficult for them to visit friends and family. Connecting to others living in the same home, then, appears to be very important in order to counteract loneliness or social isolation. Building new social relationships might facilitate the adaptation to the transition of moving in both age groups. As social approach and avoidance motives affect the success in establishing and maintaining social relationships (Gable and Berkman, 2008), they might be particularly important for successfully adapting to such transitions.

We address three aspects of transitions we expect to be associated with social approach and avoidance motives: (1) quantity of social encounters, (2) quality of social encounters, and (3) subjective well-being. We assess these three aspects on a daily level in order to reduce potential memory biases associated with retrospective reports.

### Quantity of social encounters

Gable et al. (2000) posit that positive events require active pursuit, whereas negative events occur spontaneously from time to time. As social approach motives refer to the dispositional orientation toward positive, hoped-for social incentives (McClelland, 1985), they should also lead to the active pursuit of hoped-for positive social encounters and, therefore, also to a higher frequency of interactions with other people. In line with this assumption, Gable (2006) found in two longitudinal studies with undergraduate students that social approach motivation leads to increased exposure to positive events. Thus, we expect that social approach motives lead to the active pursuit and to a higher frequency of social encounters that are experienced as positive during developmental transitions in young and older adulthood.

In contrast to social approach motives, social avoidance motives refer to the dispositional orientation away from negative, feared social incentives (McClelland, 1985). Therefore, positive social encounters should be unrelated to social avoidance motives as it is the avoidance of negative encounters that is at the core of social avoidance motives. In support of this hypothesis, Gable (2006) found no relation between social avoidance motives and the frequency of positive encounters. Note, however, that trying to avoid all potentially negative encounters can also bear the risk to miss out on positive encounters. This might be the case because the expectation of whether a social interaction might yield a positive or negative experience is likely to be biased negatively when avoidance motivation is high (see below for a more detailed discussion of this aspect). On this basis, one might even expect a negative relation between avoidance motivation and the quantity of social interactions.

### Quality of social encounters

We expect that both social approach and avoidance motives play an important role for the quality of social interactions. Previous research has shown that social approach motives are associated with the interpretation of ambiguous social information as positive (Strachman and Gable, 2006) and, more generally, with positive experiences in social encounters (Nikitin and Freund, 2010). In contrast, social avoidance motives are associated with a stronger focus on negative social information (Nikitin and Freund, 2011), with the interpretation of ambiguous social information as negative (Strachman and Gable, 2006), and, more generally, with negative experiences in social interactions (Nikitin and Freund, 2010). We expect that the focus on positive social information and the interpretation of social information as positive associated with social approach motives likely leads to positive experiences of daily social encounters. In contrast, the focus on negative social information and interpretation of social situations in a negative way associated with social avoidance motives likely leads to negative experiences in daily social encounters.

### Subjective well-being and satisfaction with the transition

Subjective well-being can be regarded as one of the central subjective indicators of successful development (for a discussion of various indicators of successful development see Freund et al., 2012). In the current studies, subjective well-being serves as an indicator of successful adaptation to the developmental transition of moving out of the parental home in young adulthood and the transition of moving into senior housing in older adulthood. To capture comprehensively subjective well-being, the current study considers four aspects of well-being: (1) subjective physical well-being, (2) emotional well-being, (3) (the absence of) feelings of loneliness. (4) Satisfaction with the transition serves as an additional indicator of successfully mastering the move in young and old adulthood. We expect that social approach motives are associated positively with subjective well-being, whereas social avoidance motives are associated negatively with subjective well-being during developmental transitions in young and older adulthood.

## The Role of Age for Transitions

Age plays an important role in social experience and behavior (e.g., Carstensen et al., 1999; Antonucci et al., 2010). Social-network size is reduced in old age due to focusing on the closest social partners such as family and confidants (e.g., Lang et al., 1998). Thus, compared to younger adults we expect older adults to pursue fewer social encounters, particularly with persons who are not close.

In the context of conflicts with social partners, older adults seem to avoid conflict and negative behavior more frequently than younger age groups (Carstensen et al., 1995), and they profit more from avoiding conflict than younger adults (Charles et al., 2009). Thus, older adults are expected to report fewer negative (own and others’) behaviors than younger adults, as they are particularly motivated to avoid conflict (Diamond et al., 2010).

Does age moderate the association between dispositions and their outcomes during a transition? There are good reasons for expecting either stability, increase, or decrease in the impact of social approach and avoidance motives on mastering the transition of a move across adulthood. Speaking for a decrease, Ready and Robinson (2008) found that extraversion and neuroticism (which are associated with social approach and avoidance motives, respectively; Nikitin and Freund, 2010) were more predictive of emotional experience among younger than older adults. The authors concluded that emotion regulation, self-acceptance, and adjustment (factors that are high among older adults; Ryff and Keyes, 1995) might be more important for successful development in older adults than dispositions. However, there is also empirical evidence for an increase in the effects of dispositions over time. In three studies by Impett et al. (2010), participants with approach relationship goals – that are closely related to social approach motives (Gable, 2006) – experienced increases in relationship satisfaction and commitment over a 3-month period. In contrast, the number of avoidance goals – that are closely related to social avoidance motives (Gable, 2006) – was associated with decreases in relationship satisfaction over the same period of time. This finding might be explained by cumulative effects of approach and avoidance motives with positive expectations begetting positive responses that lead to more positive expectations, etc., and vice versa for negative expectations (Downey et al., 1998). Finally, there are also theoretical reasons and empirical support for assuming stability in the effect of social dispositions across adulthood. On an empirical level, despite the often dramatic changes in people’s lives and their environments that occur across adulthood, personality such as the “Big Five” remain relatively stable over time (e.g., Terracciano et al., 2010). One of the explanations for this finding might lie in a process often called cumulative continuity (Caspi et al., 2005). Cumulative continuity, similar to Neugarten’s notion of the institutionalization of personality in adulthood (Neugarten, 1964), describes the phenomenon that personality traits and the (social) environment interact in a way that tends to stabilize the relation between the two. One of the factors contributing to cumulative continuity is the process of “niche-building,” i.e., people create or seek environments that correspond to their traits. Moreover, the social environment over time builds expectations regarding a person’s behavior and sanctions positively when expectations are fulfilled. This, in turn, makes it more likely that people will behave in accordance with social expectations. Over time, then, both the person and the social environment will show a pattern of a stable interaction with each other. In line with the stability hypothesis, Isaacowitz (2005) found no age-related differences in the effect of dispositional optimism on depression and life satisfaction. More closely related to the current topic of social motives, Nikitin and Freund (2011) found the same effect of avoidance motives on gaze behavior to positive (happy) and negative (angry) facial expressions in younger and older adults.

## The Role of Time for Transitions

Does the influence of social approach and avoidance motivation change over the course of a transition? Caspi and Moffitt (1993) propose that individual differences tend to be magnified when persons experience profound discontinuities in their lives. Preexisting cognitive schemas exert a powerful and pervasive influence on our interpretation of new experiences by helping us categorize and organize the changing events around us (see also Weiss et al., 2012). Caspi and Moffitt conclude that individual differences are most likely accentuated during transitions into new situations that are characterized by unpredictability, when there is a press to behave but no information about how to behave in a socially adequate way. In such situations, persons attempt to assimilate the new events into existing cognitive and action structures.

Caspi and Moffitt further claim that the accentuation of individual differences can be operationalized either quantitatively or qualitatively. Quantitative accentuation is related to differences between individuals on a single trait dimension (i.e., mean differences). In other words, transitions should expand the range of scores within a sample. We tested the second–stronger–version of the accentuation hypothesis, the qualitative hypothesis. This version of the accentuation hypothesis states that trait-related behaviors, cognitions, and emotions should be exhibited more frequently, the less familiar the situation. Thus, we hypothesize that social approach and avoidance motives will be most predictive for social experience and behavior directly after the transition. The longer the time from the transition, the fewer unpredictable and ambiguous situations a person encounters. Therefore, with increasing time from the transition, the effects of social approach and avoidance motives should decrease.

To summarize, we hypothesize:

1. Outcomes of social approach and avoidance motives:
  1a. Social approach motives are positively associated with the quantity and the quality of social contacts as well as subjective well-being.
  1b. Social avoidance motives are unrelated or negatively related to the quantity of social contacts and negatively related to their quality and to subjective well-being.
2. Age-related differences:
  2a. Older adults report fewer social contacts than younger adults, particularly fewer negative interactions.
  2b. On the basis of Neugarten’s notion of institutionalization of personality, we expect stability in the association between social motives and their outcomes. The stability hypothesis will be tested against the increase and decrease hypothesis.
3. Time during the transition:
  The impact of social approach and avoidance motives is strongest in the transition phase and decreases over time.

This set of hypotheses was tested in two studies targeting developmental transitions in young and older adults. Study 1 investigated the developmental transition of moving out of the parental home into shared housing when starting to study at a university. Study 2 was concerned with moving out of a private household into senior housing facilities in older adulthood. As both studies follow a similar method, we present the sample, procedure, and instruments within one combined Section “Materials and Methods.”

## Materials and Methods

### Study 1: Younger sample

The diary data reported in the present article stem from university students (*N* = 93, 75% females) of a wide range of courses. The age was *M* = 21.59 years (SD = 2.11, range 18–30). The majority of the participants (81%) were German, 9.6% were Swiss, and 9.8% reported other European nationalities, but with very good knowledge of the German language. Forty-seven percent of the sample reported to be in a steady partnership, and one person had a child. One-third (37.6%) of the students worked part-time. Participants were recruited via online advertisements at various German and Swiss universities.

All participants had recently moved out of their parental home to a shared apartment. Immediately before or recently after the move, participants filled out a questionnaire assessing social approach and avoidance motivation (and some other constructs that are not reported here). Participants had moved on average 27.98 days (SD = 99.45) before they completed the first questionnaire. Directly after the move or up to 1 week after filling out the first questionnaire, the diary subsample reported daily social experience and behavior over a period of 2 weeks (i.e., a total of 14 diaries). Seventy-one percent of all questionnaires were completed. Each person completed *M* = 9.98 (SD = 4.64) diaries.

### Study 2: Older sample

The majority of the older adults (*N* = 69, *M* = 76.95 years, SD = 7.15, range 61–90; 61% females) who participated in the study were Swiss (95.6%), one was German, and two had other European nationalities, but with very good knowledge of the German language. Regarding relationship status, 33.3% of the participants were married or in a long-term relationship, 31.9% were widowed, 15.9% divorced, and 18.8% single. The majority of the older adults had children (71%). Regarding education, 27.5% held a university degree or professional training, 27.5% had a high school degree, 15.9% had completed an apprenticeship, and 13% obligatory school (10 years of school). All of the older participants were retirees.

All participants had moved into senior housing up to 5 years ago. In Switzerland, senior housing arrangements comprise apartments adapted to the health-related needs of elderly people (e.g., no stairs, large doors, emergency buttons). Furthermore, senior housing arrangements offer services such as nursing care or delivered meals, communal facilities, and sometimes organized leisure activities. The residents live independently but can require specific services if needed. We asked directors of several urban and rural senior housing facilities in Switzerland for permission to recruit participants. Participants were then recruited via an invitation letter, followed by a telephone to explain the study. Participants who were interested in the study filled out a questionnaire on their social motivation (and other constructs not relevant in the present context). Approximately a week later, they started the diary part of the study with 14 consecutive questionnaires. They completed a total of 904 questionnaires (*M* = 13.5, SD = 1.41), that means, 93.6% of all questionnaires were completed. Participants had moved *M* = 2.56 years (SD = 1.36) before the beginning of the study. Thus, the move in the older sample dated back much longer than in the younger sample, *t*(158) = −17.16, *p* < 0.001. Effects of time since move and age will be reported for all analyses.

### Measures

If not noted otherwise, responses were given on a seven-point Likert scale ranging from 0 (strongly disagree) to 6 (strongly agree).

#### Age

Participants reported their birthdate. Age was calculated as a difference between their birthdate and the first measurement time point (assessing social approach and avoidance motives).

#### Social approach and avoidance motives

We used the Affiliation Tendency and Sensitivity to Rejection Scale (Mehrabian, 1970; German version in Sokolowski, 1986) to assess social approach and avoidance motives. These scales consist of 50 self-descriptive statements portraying typical social behavior and experience. The affiliation tendency subscale (25 items; sample item: “I like to make as many friends as I can”) was used to measure social approach motives, the rejection sensitivity subscale (25 items; sample item: “I prefer not to go to a place if I know that some of the people who will be there don’t like me”) was used to measure social avoidance motives.

Although widely used in younger samples, to our knowledge this was the first time this questionnaire was used with older adults. Reliability analyses in the older sample revealed three items that loaded negatively on the rejection sensitivity scale and one item that loaded negatively on the affiliation tendency scale in the older sample. After excluding these items, the internal consistency was Cronbach’s α = 0.71 for approach motivation (*M* = 3.24, SD = 0.71) and α = 0.77 for avoidance motivation (*M* = 3.21, SD = 0.75). Approach and avoidance motivation were not significantly correlated (*r* = −0.17, *p* = 0.16). Using the same items, for the younger sample the internal consistency was Cronbach’s α = 0.79 for approach motivation (*M* = 3.56, SD = 0.74) and α = 0.83 for avoidance motivation (*M* = 3.14, SD = 0.71). The two constructs were again not significantly correlated (*r* = −0.12, *p* = 0.27). Older participants were significantly less approach motivated than younger participants, *t*(160) = 2.79, *p* = 0.006, whereas social avoidance motivation did not significantly differ between the two groups, *t*(160) < −1, *p* = 0.56.

#### Quantity of daily social encounters

The assessment of the quantity of social encounters was based on the Rochester Interaction Record (Reis and Wheeler, 1991). The overall number of interactions was assessed with the question “In the last 24 h, with how many people did you have an interaction (including conversations per phone, skype, internet chat) that lasted longer than 10 min?” Social interaction was defined as any encounter with one or more other person(s) in which the persons interacted with each another. The mere presence of another person was not included in this definition. To assess the closeness of the interaction partners, we asked how many of these persons were “very close,” “close,” “less close,” and “not at all close.” Participants reported the number of very close, close, less close, and not at all close persons with whom they interacted on the particular day. Further, participants were asked if they actively pursued social contacts with other persons (“Did you seek contact to very close/close/less close/not at all close persons?”) and if they made any new acquaintances (0 = no, 1 = yes).

#### Quality of daily social encounters

Three aspects of the quality of social encounters were assessed: (a) pleasantness of social interactions, (b) positive and negative own and others’ behavior, (c) negative cognitions about social relationships and interactions.

##### Pleasantness of social interactions

After participants reported how many interactions they had had in the last 24 h, they were asked how positive or negative the majority of these interactions was (0 = very negative, 6 = very positive). The same question was asked for the new acquaintance (“If you made a new acquaintance, was this contact positive or negative?”).

##### Positive and negative own and others’ behavior

Four items assessed own positive behavior (“I praised or complimented someone,” “I was affectionate with someone,” “I was sympathetic to someone,” “I showed someone my affection”), five items own negative behavior (“I ignored someone,” “I affronted or hurt someone,” “I criticized someone,” “I complained to somebody,” “I was ruthless or egoistic”). The same items assessed positive and negative behavior of others. Own and others’ positive behaviors were highly positively correlated on the daily level (correlations from *r* = 0.70 to *r* = 0.88, all *p*_s_ < 0.001, *M*_r_ = 0.83, SD_r_ = 0.06 for younger adults; correlations from *r* = 0.76 to *r* = 0.92, *p*_s_ < 0.001, *M*_r_ = 0.86, SD_r_ = 0.04 for older adults). The same was true for own and others’ negative behavior (correlations from *r* = 0.51 to *r* = 0.80, all *p*_s_ < 0.001, *M*_r_ = 0.66, SD_r_ = 0.09 for younger adults; correlations from *r* = 0.43 to *r* = 0.73, all *p*_s_ < 0.001, *M*_r_ = 0.60, SD_r_ = 0.10 for older adults). Additionally, own and others’ behavior did not lead to different results in the subsequent multilevel analyses. Thus, we combined own and others’ positive behavior and own and others’ negative behavior into one composite score. Positive and negative behaviors were not significantly correlated (correlations from *r* = −0.19 to *r* = 0.25, all *p*_s_ > 0.05, *M*_r_ = 0.12, SD_r_ = 0.10 for younger adults; correlations from *r* = −0.13 to *r* = 0.21, all *p*_s_ > 0.05, *M*_r_ = 0.05, SD_r_ = 0.10 for older adults).

##### Negative cognitions about social relationships and interactions

We assessed negative cognitions about social relationships and interactions using seven items from the Fear of Negative Evaluations Scale (Leary, 1983): “I was afraid to disappoint someone,” “I felt loved/liked” (reversed), “I was afraid to anger someone,” “I was worried about my relationships,” “I was not sure if my presence is desired,” “I felt rejected,” and “I felt accepted as I am” (reversed).

#### Daily subjective well-being

Four aspects of subjective well-being were assessed: physical well-being, emotional well-being, loneliness, satisfaction with the move.

Physical well-being was assessed using a single item (“Did you felt physically well?” 0 = not at all to 6 = very). Emotional well-being was assessed with the short version of the Multidimensional Mood Questionnaire (MDMF; Steyer et al., 1997). The short version of the MDMF consists of 12 adjectives that can be aggregated into a score reflecting emotional well-being. Loneliness was measured with four items from the UCLA Loneliness Scale (Russell, 1996; German version Döring and Bortz, 1993): “I had people that really understand me” (reversed), “I felt excluded,” “There were people that I could turn to” (reversed), and “I felt alone.”

Finally, satisfaction with the move was assessed with two questions: “In the last 24 h, were there moments when you regretted having moved?” (reversed) and “In the last 24 h, were there moments when you were happy about the move?”

Descriptive statistics (M, SD, α) of all diary constructs are reported in Table 1. Bivariate correlations of all assessed constructs aggregated over the 2 weeks of the diary phase are presented in Table 2.

**Table 1:** **Descriptive statistics of the diary data**.

	Young					Old				
	*N*	*M*_Mean_ (SD_Mean_)	*M*_SD_ (SD_SD_)	α		*N*	*M*_Mean_ (SD_Mean_)	*M*_SD_ (SD_SD_)	α	
				*M* (SD)	Min, max				*M* (SD)	Min, max
**QUANTITY OF SOCIAL ENCOUNTERS**										
Interaction number	53–78	6.80 (0.45)	5.52 (1.04)			60–67	3.97 (0.52)	4.15 (1.15)		
Interaction frequency with very close persons	49–76	2.08 (0.32)	1.52 (0.44)			61–67	1.11 (0.17)	1.34 (0.21)		
Interaction frequency with close persons	38–64	2.09 (0.28)	2.10 (0.66)			60–67	0.91 (0.15)	1.78 (0.39)		
Interaction frequency with less close persons	31–67	2.39 (0.31)	2.64 (0.79)			61–67	1.24 (0.25)	2.33 (0.64)		
Interaction frequency with not close persons	29–51	2.55 (0.56)	3.34 (1.39)			60–66	0.70 (0.26)	1.82 (0.95)		
Active approach of very close persons	53–78	3.88 (0.13)	1.96 (0.11)			47–58	3.62 (0.37)	2.23 (0.15)		
Active approach of close persons	53–78	2.81 (0.28)	2.12 (0.15)			35–45	2.66 (0.42)	2.16 (0.11)		
Active approach of less close persons	53–78	2.13 (0.30)	1.91 (0.11)			31–47	1.84 (0.30)	1.80 (0.19)		
Active approach of not close persons	53–78	1.36 (0.18)	1.82 (0.15)			31–40	1.10 (0.29)	1.62 (0.26)		
New social contacts	53–78	0.30 (0.05)	0.46 (0.02)			60–68	0.13 (0.04)	0.33 (0.04)		
**QUALITY OF SOCIAL ENCOUNTERS**										
Interaction pleasant	52–77	4.70 (0.15)	1.09 (0.16)			51–61	4.95 (0.16)	1.09 (0.16)		
Interaction new pleasant	19–30	4.10 (0.37)	1.75 (0.31)			4–12	4.55 (0.37)	1.17 (0.44)		
Positive behavior	53–77	3.38 (0.24)	1.54 (0.11)	0.89 (0.02)	0.86,0.93	58–67	3.01 (0.14)	1.68 (0.09)	0.73 (0.03)	0.68,0.77
Negative behavior	53–77	1.01 (0.17)	1.00 (0.13)	0.85 (0.04)	0.77,0.89	58–66	0.27 (0.06)	0.59 (0.08)	0.71 (0.05)	0.64,0.78
Negative cognitions	53–76	1.39 (0.15)	1.00 (0.09)	0.71 (0.06)	0.58,0.77	58–66	0.86 (0.05)	0.79 (0.07)	0.72 (0.06)	0.64,0.84
**SUBJECTIVE WELL-BEING**										
Physical well-being	53–76	3.57 (0.11)	1.60 (0.09)			61–67	4.13 (0.16)	1.46 (0.10)		
Emotional well-being	53–76	3.67 (0.15)	1.05 (0.08)	0.87 (0.02)	0.84,0.92	59–68	4.41 (0.10)	1.03 (0.08)	0.90 (0.02)	0.86,0.92
Loneliness	53–76	1.35 (0.12)	1.17 (0.09)	0.75 (0.07)	0.55,0.82	60–66	1.12 (0.11)	1.03 (0.10)	0.60 (0.05)	0.40,0.73
Regret about the move	53–76	0.89 (0.17)	1.38 (0.20)			62–68	0.27 (0.06)	0.86 (0.14)		
Happiness with the move	53–76	3.37 (0.19)	2.04 (0.12)			61–68	5.22 (0.12)	1.47 (0.17)		

**Table 2:** **Bivariate correlations among the diary constructs (at aggregated daily level)**.

	1	2	3	4	5	6	7	8	9	10	11	12	13	14	15	16	17	18	19	20
1. Interaction number		**0.39**	**0.58**	**0.65**	**0.64**	0.04	**0.24**	**0.22**	**0.22**	**0.31**	0.01	**0.16**	**0.27**	**0.08**	−0.05	0.03	**0.09**	−**0.19**	−**0.12**	**0.13**
2. Interaction frequency with very close persons	**0.33**		**0.30**	0.07	−0.04	**0.21**	**0.11**	−0.06	−0.06	0.03	**0.14**	0.02	**0.25**	**0.07**	−**0.14**	0.00	**0.14**	−**0.24**	−0.06	**0.15**
3. Interaction frequency with close persons	**0.54**	0.06		**0.32**	0.03	0.02	**0.25**	**0.09**	−0.01	**0.17**	**0.09**	0.06	**0.19**	0.00	−0.03	0.04	**0.13**	−**0.16**	−0.07	**0.17**
4. Interaction frequency with less close persons	**0.67**	−0.02	**0.14**		**0.13**	−**0.10**	0.06	**0.21**	−0.01	**0.15**	−0.02	**0.15**	**0.14**	−0.01	−0.02	−0.05	0.01	−**0.09**	−0.04	**0.11**
5. Interaction frequency with not close persons	**0.61**	−0.03	**0.12**	**0.13**		−0.07	−0.04	0.08	**0.28**	**0.22**	−**0.10**	0.12	0.03	**0.12**	0.06	0.02	−0.01	0.03	−0.03	−0.04
6. Active approach of very close persons	**0.16**	**0.34**	**0.14**	−0.03	−0.03		**0.39**	**0.17**	**0.10**	−0.01	**0.16**	−0.04	**0.35**	**0.11**	−**0.10**	0.01	**0.10**	−**0.23**	−0.01	0.04
7. Active approach of close persons	**0.16**	**0.19**	**0.32**	0.02	−**0.10**	**0.55**		**0.35**	**0.11**	**0.13**	**0.08**	0.10	**0.21**	**0.09**	−0.01	−0.06	0.01	−**0.11**	−0.03	**0.13**
8. Active approach of less close persons	**0.13**	0.04	0.04	**0.20**	−0.03	**0.26**	**0.38**		**0.31**	**0.12**	**0.11**	**0.16**	**0.13**	0.03	−0.04	0.02	0.03	−**0.09**	0.00	**0.09**
9. Active approach of not close persons	**0.16**	−0.02	0.08	0.04	**0.23**	0.09	**0.25**	**0.41**		**0.29**	**0.11**	**0.28**	**0.11**	0.02	0.01	**0.11**	**0.08**	−0.03	−0.06	0.03
10. New social contacts	**0.26**	0.03	**0.17**	**0.15**	**0.23**	0.05	0.00	**0.12**	**0.21**		0.05	**0.57**	**0.14**	0.03	0.00	0.05	0.03	−0.03	−0.04	**0.11**
11. Interaction pleasant	**0.08**	**0.23**	**0.09**	−0.05	−0.02	**0.28**	0.02	−**0.09**	0.04	0.02		**0.41**	**0.28**	−**0.25**	−**0.38**	**0.29**	**0.40**	−**0.43**	−**0.24**	**0.19**
12. Interaction new pleasant	0.10	**0.25**	0.17	0.01	−0.04	0.09	0.10	−0.02	−0.01	−0.06	**0.31**		0.06	−0.06	−0.12	**0.11**	**0.14**	−**0.15**	−0.09	**0.19**
13. Positive behavior	**0.19**	**0.37**	**0.19**	0.00	0.00	**0.56**	**0.29**	**0.16**	0.08	**0.13**	**0.40**	**0.32**		**0.13**	−**0.24**	**0.17**	**0.26**	−**0.51**	−**0.12**	**0.24**
14. Negative behavior	0.00	−0.01	−0.03	0.04	0.02	0.06	**0.09**	**0.11**	0.08	**0.10**	−**0.32**	−**0.37**	0.05		**0.42**	−**0.19**	−**0.27**	**0.19**	**0.24**	−**0.08**
15. Negative cognitions about interactions	−**0.07**	−**0.14**	−**0.09**	0.01	0.04	−**0.12**	−0.01	**0.11**	0.08	0.03	−**0.44**	−**0.33**	−**0.35**	**0.50**		−**0.34**	−**0.59**	**0.62**	**0.34**	−**0.21**
16. Physical well-being	0.04	0.04	0.01	−0.01	0.03	0.02	−0.06	−**0.11**	−0.05	0.01	**0.27**	−0.02	**0.17**	−**0.19**	−**0.36**		**0.65**	−**0.39**	−**0.20**	**0.20**
17. Emotional well-being	0.06	0.02	**0.10**	−0.01	0.05	0.07	−0.07	−**0.16**	−**0.12**	0.00	**0.38**	0.18	**0.24**	−**0.30**	−**0.55**	**0.63**		−**0.58**	−**0.30**	**0.32**
18. Loneliness	−**0.18**	−**0.27**	−**0.16**	−0.01	−0.04	−**0.36**	−**0.18**	0.01	0.00	−0.06	−**0.52**	−**0.43**	−**0.58**	**0.23**	**0.49**	−**0.26**	−**0.44**		**0.29**	−**0.30**
19. Regret about the move	**0.07**	0.01	−0.03	**0.11**	0.04	−0.02	0.05	**0.15**	**0.16**	0.03	−0.06	0.06	−0.02	**0.15**	**0.24**	−**0.13**	−**0.34**	**0.14**		−**0.20**
20. Happiness with the move	**0.08**	0.05	**0.13**	0.05	0.06	0.04	−0.07	−**0.19**	−**0.11**	−0.04	**0.10**	0.08	**0.19**	−**0.07**	−**0.17**	**0.15**	**0.33**	−**0.17**	−**0.42**	

*Correlations of the daily constructs for young adults are in the right upper part of the table, for older adults in the left lower part of the table. Correlations that are significant at the *p* < 0.05 or lower levels are presented in bold*.

### Data-analytical plan

To test the hypotheses of age and social motives as predictors of daily experience and behavior, we ran multilevel analyses with age, time since move, and approach and avoidance motives (centered) as predictors. Age was introduced as a dummy variable with 0 (young adults) and 1 (older adults). We analyzed the data with the linear mixed-models procedure (with Maximum Likelihood Method for deriving the estimates) using SPSS Statistics Version 20 with day of the diary (1–14) as level 1 variable and participants as level 2 variable. As the day of the diary did not show any linear or curvilinear trend in almost all of the analyzed variables over the 2 weeks, we excluded it from the analyses. Results of the multilevel analyses are presented in Table 3. As we ran 24 tests to test the hypotheses, we used Sidak’s correction (Sidak, 1967) to adjust the alpha level. Results that are significant on this corrected *p-*level (*p* < 0.0021) are presented in bold in Table 3.

**Table 3:** **Estimates of fixed effects and random parameters for models of the predictors of daily social behavior and experience**.

	Fixed effects					Random parameters	
	Intercept	Time	Age	Approach	Avoidance	Residual	Intercept
**QUANTITY OF SOCIAL ENCOUNTERS**							
Interaction Number	6.50*** (0.39)	0.25 (0.31)	−3.25** (1.04)	1.39*** (0.36)	0.09 (0.35)	15.26*** (0.53)	8.11*** (1.11)
Interaction freq. with very close persons	2.03*** (0.11)	−0.08 (0.08)	−0.59* (0.28)	0.52*** (0.10)	0.01 (0.09)	1.55*** (0.06)	0.54*** (0.08)
Interaction freq. with close persons	1.94*** (0.14)	0.02 (0.10)	−1.00** (0.35)	0.62*** (0.12)	−0.02 (0.12)	2.40*** (0.09)	0.80*** (0.12)
Interaction freq. with less close persons	2.28*** (0.19)	−0.01 (0.14)	−0.98* (0.47)	0.35* (0.17)	0.17 (0.16)	4.71*** (0.17)	1.51*** (0.23)
Interaction freq. with not close persons	2.46*** (0.13)	0.07 (0.13)	−2.05*** (0.44)	−0.20 (0.16)	−0.16 (0.15)	5.92*** (0.24)	1.04*** (0.22)
Active approach of very close persons	3.68*** (0.17)	0.21 (0.13)	−0.85 (0.45)	0.66*** (0.16)	0.25 (0.15)	2.54*** (0.09)	1.49*** (0.20)
Active approach of close persons	2.68*** (0.16)	0.25 (0.13)	−0.74 (0.44)	0.67*** (0.15)	0.17 (0.14)	3.00*** (0.12)	1.26*** (0.19)
Active approach of less close persons	2.04*** (0.14)	0.17 (0.12)	−0.64 (0.39)	0.36** (0.13)	0.09 (0.12)	2.55*** (0.10)	0.92*** (0.14)
Active approach of not close persons	1.21*** (0.13)	0.26* (0.10)	−0.90* (0.35)	0.01 (0.12)	0.02 (0.11)	2.40*** (0.09)	0.68*** (0.11)
New social contacts	0.27*** (0.02)	0.02 (0.02)	−0.21*** (0.05)	0.07*** (0.02)	−0.01 (0.02)	0.15*** (0.01)	0.01*** (0.00)
**QUALITY OF SOCIAL ENCOUNTERS**							
Interaction pleasant	4.63*** (0.09)	0.02 (0.07)	0.32 (0.23)	0.28** (0.08)	−0.23** (0.08)	0.74*** (0.03)	0.40*** (0.05)
Interaction new pleasant	4.17*** (0.15)	0.00 (0.13)	0.40 (0.42)	0.11 (0.15)	−0.31* (0.13)	2.01*** (0.15)	0.50*** (0.13)
Positive behavior	3.27*** (0.15)	0.09 (0.12)	−0.44 (0.39)	0.66*** (0.14)	0.02 (0.13)	1.05*** (0.04)	1.27*** (0.16)
Negative behavior	1.02*** (0.07)	0.06 (0.06)	−0.96*** (0.19)	0.01 (0.07)	0.11 (0.06)	0.44*** (0.02)	0.28*** (0.04)
Negative cognitions about interactions	1.50*** (0.08)	0.03 (0.06)	−0.82*** (0.20)	−0.29*** (0.07)	0.37*** (0.07)	0.41*** (0.01)	0.33*** (0.04)
**SUBJECTIVE WELL-BEING**							
Physical well-being	3.57*** (0.14)	0.03 (0.11)	0.47 (0.21)	−0.00 (0.13)	−0.43** (0.12)	1.17*** (0.04)	1.08*** (0.14)
Emotional well-being	3.60*** (0.09)	−0.03 (0.07)	0.98*** (0.24)	0.26** (0.08)	−0.43*** (0.08)	0.48*** (0.02)	0.47*** (0.06)
Loneliness	1.47*** (0.10)	0.00 (0.08)	−0.42 (0.25)	−0.44*** (0.09)	0.24** (0.09)	0.61*** (0.02)	0.51*** (0.07)
Regret about the move	0.77*** (0.10)	0.16* (0.08)	−1.07*** (0.27)	−0.08 (0.09)	0.18* (0.09)	0.76*** (0.03)	0.58*** (0.07)
Happiness with the move	3.37*** (0.18)	0.07 (0.14)	1.64** (0.47)	0.43* (0.16)	−0.11 (0.16)	1.19*** (0.04)	1.87*** (0.23)

Interaction freq. = Interaction frequency. ***p < 0.001. **p < 0.01. *p < 0.05. Standard errors are in parentheses. Results in bold are significant after Sidak’s adjustment (adjustment of the significance level from *p* < 0.05 to *p* < 0.0021 for the 24 tests). The models presented in the table are with random intercepts (the models with the best fit).

To test if age moderated the relationship between social motives and daily outcomes, we included the interaction of age and social approach and avoidance motives, respectively, in the multilevel models:

$$\begin{array}{l} \text{Outcome }{\text{variable}}_{ij}=b_{0j}+b_{1}\text{ Time since }{\text{move}}_{ij}+b_{2}{\text{Age}}_{ij}\\ \;+b_{3}\;{\text{Approach motives}}_{ij}\\ \;+b_{4}\text{ Avoidance }{\text{motives}}_{ij}\\ \;+b_{5}\;{\left(\text{Age}\times \text{Approach motives}\right)}_{ij}\\ \;+b_{6}\;{\left(\text{Age}\times \text{Avoidance motives}\right)}_{ij}+\varepsilon_{ij}\\ \;b_{0j}=b_{0}+u_{0j} \end{array}$$

Significant interactions of age and motives were probed by using a subgroup-analysis approach, where the data are split into two age groups (young and old) and the analyses are repeated on these subgroups (for a discussion on the limits of this approach see Newsom et al., 2003). Significant fixed effects of social motives for daily outcomes in the older but not (or reduced) in the younger group would support the increase hypothesis, significant fixed effects of social motives for daily outcomes in the younger but not (or reduced) in the older group would support the decrease hypothesis. No significant interactions would support the stability hypothesis.

We also tested the hypothesis that the effects of social approach and avoidance motives are strongest in the transition phase and decrease with the time since the move. To test this hypothesis, we included the interaction of time from the move and social approach and avoidance motives, respectively, in the multilevel models. We again split the file into two groups, a group whose date of move was less than 3 months ago (*n* = 78, 77 young persons) and a group that moved more than 3 months ago (*n* = 82, 16 young persons). This variable is included in the multilevel models as a dummy variable (0 = less than 3 months after the move, 1 = more than 3 months after the move). Because this variable is almost perfectly confounded with age and, in this context, we were interested in time from move (and not age), we controlled for age in these models:

$$\begin{array}{l} {\text{Outcome variable}}_{ij}=b_{\text{0}j}+b_{\text{1}}{\text{ Age}}_{ij}+b_{\text{2}}{\text{ Time since move}}_{ij}\\ \;+b_{\text{3}}{\text{ Approach motives}}_{ij}\\ \;+b_{\text{4}}{\text{ Avoidance motives}}_{ij}\\ \;+b_{\text{5}}\;(\text{Time since move}\\ \;{\times \text{Approach motives})}_{ij}\\ \;+b_{\text{6}}\;(\text{Time since move}\\ \;{\times \text{Avoidance motives})}_{ij}+\varepsilon_{ij}\\ \;b_{\text{0}j}=b_{\text{0}}+u_{\text{0}j} \end{array}$$

Significant interactions of age and motives were again probed by using a subgroup-analysis approach, where the data are split into two time groups (less or more than 3 months since the move) and the analyses were repeated on these subgroups. Significant fixed effects of social motives for daily outcomes in the more recent but not (or reduced) in the less recent group would support the hypothesis that the impact of social approach and avoidance motives is strongest in the transition phase and decreases over time.

## Results

### Outcomes of social approach and avoidance motives

#### Social approach motives and quantity and quality of social encounters

Social approach motives were positively associated with the number of social interactions, *t*(158.87) = 3.84, *p* < 0.001, the frequency of interacting with very close, *t*(147.17) = 5.33, *p* < 0.001, and close persons, *t*(153.36) = 5.12, *p* < 0.001, the active pursuit of interactions with very close, *t*(163.02) = 4.22, *p* < 0.001, and close persons, *t*(159.73) = 4.49, *p* < 0.001, and the frequency of entering new social relations, *t*(148.07) = 3.95, *p* < 0.001. Unexpectedly, social approach motives did not predict the active pursuit of and contact frequency with persons to whom participants did not feel close (see Table 3, upper part).

The hypotheses regarding the effects of social approach motives on the quality of social encounters were mostly supported (see Table 3, middle part). Social approach motives were related to positive behavior in social encounters, *t*(161.70) = 4.84, *p* < 0.001. Further, higher social approach motives were associated with more pleasant social encounters, *t*(146.85) = 3.47, *p* < 0.001, and fewer negative cognitions about social relationships, *t*(150.91) = −4.12, *p* < 0.001. Unexpectedly, social approach motives were unrelated to the pleasantness of new social interactions.

We hypothesized that social approach motives predict high subjective well-being. Results of the multilevel analyses partly supported this hypothesis (see Table 3, lower part). Social approach motives were negatively related to loneliness, *t*(155.63) = −4.96, *p* < 0.001, but unrelated to physical well-being and regretting the move.

#### Social avoidance motives and quantity and quality of social encounters

The results support the hypothesis that there is no relationship between social avoidance motives and quantity of social encounters (see Table 3, upper part). In addition to the quantity of social encounters, social avoidance motives were associated with negative cognitions about social interactions, *t*(140.50) = 5.34, *p* < 0.001. Higher social avoidance motives were associated with more negative cognitions about social interactions than lower social avoidance motives (see Table 3, middle part). Unexpectedly, social avoidance motives were unrelated to negative social behavior. There were no other associations with daily behavior.

We hypothesized that social avoidance motives are negatively associated with subjective well-being. Results of the multilevel analyses partly supported this hypothesis (see Table 3, lower part). Social avoidance motives were associated with lower physical, *t*(149.88.30) = −3.52, *p* = 0.001, and emotional, *t*(149.63) = −5.24, *p* < 0.001, well-being but unrelated to happiness with the move.

### Age-related differences

#### Age and quantity and quality of social interactions

As hypothesized, age was negatively related to the number of daily interactions, *t*(148.13) = −2.13, *p* = 0.002 (see Table 3, upper part), particularly with persons to whom participants did not feel close, *t*(118.69) = −4.64, *p* < 0.001. Older adults also reported entering fewer new social relationships than younger adults did, *t*(145.00) = −4.06, *p* < 0.001. Age was unrelated to the active approach of social partners.

Confirming hypotheses, older adults did not report more positive behaviors than younger adults, but they reported fewer negative behaviors in their daily interactions, *t*(142.08) = −5.07, *p* < 0.001 (see Table 3, middle part). Further, older adults experienced the majority of their daily interactions and new social interactions equally positive as younger adults. Older adults reported fewer negative cognitions about their social relationships than younger adults did, *t*(142.46) = 3.98, *p* < 0.001.

#### Age as a moderator of the association between social motives and daily outcomes

One important question of this study was if the effects of social approach and avoidance motives differ between young and older adults. We introduced three alternative hypotheses of (1) stronger effects with age, (2) weaker effects with age, and (3) no age-related differences. To test theses hypotheses, we included the interaction of age and social approach and avoidance motives, respectively, in the multilevel models reported in Table 3. None of these models fitted the data significantly better than the main-effect models. Thus, this study provides further support for the stability hypothesis stating that the effects of approach and avoidance motives do not differ across age groups.

### Time since move as a moderator of the association between social motives and daily outcomes

We also tested the hypothesis that the effects of social approach and avoidance motives are strongest in the transition phase and decrease with the time since the move. Results of the multilevel analyses revealed that the effect of social approach and avoidance motives on the quantity, quality, and active pursuit of social interactions was not moderated by time. One exception was a change over time in the effect of social approach motives on the active pursuit of interactions with close social partners. Up to 3 months, social approach motives were positively related to the active pursuit of social interactions, *b* = 0.95, SE*b* = 0.20, *t*(78.71) = 4.74, *p* < 0.001. In the group that moved more than 3 months ago, the association was still positive but significantly weaker, *b* = 0.33, SE*b* = 0.20, *t*(73.21) < 1, *p* = 0.62, $\chi_{\text{diff}}^{2}(2)=6.61$, *p* < 0.05. Apart from this exception, the effect of social motives on the quantity, quality, and active pursuit of social interactions seems quite stable.

In contrast, the effects of social approach and avoidance motives on variables related to subjective well-being differed by time since the move. The interactions of social avoidance motives and the time since the move were significant for physical well-being, *F*(1, 150.81) = 6.70, *p* < 0.05, emotional well-being, *F*(1, 150.24) = 4.69, *p* < 0.05, and happiness with the move, *F*(1, 152.58) = 10.11, *p* < 0.01. Regarding social approach motives, only the effect on loneliness was significantly moderated by time, *F*(1, 156.98) = 5.24, *p* < 0.05. All of these models had a better fit than the main-effect models (as measured by the difference in −2*log likelihood and number of parameters): for physical well-being, $\chi_{\text{diff}}^{2}(2)=6.52$, *p* < 0.05, for emotional well-being, $\chi_{\text{diff}}^{\text{2}}\left(2\right)=5\text{.99},\;p<\text{0}\text{.05,}$ for loneliness, $\chi_{\text{diff}}^{\text{2}}\left(2\right)=10\text{.26},\;p<\text{0}\text{.01,}$ and for happiness with the move, $\chi_{\text{diff}}^{\text{2}}\left(2\right)=10\text{.98},\;p<\text{0}\text{.01}$.

To better understand these interaction effects, we reran the analyses separately for the two time-from-move groups. The results for social avoidance motives were consistent with the hypothesis. The effect of social avoidance motives was lower in the group that had moved more than 3 months ago compared to the group that had moved less than 3 months ago for all outcome variables: physical well-being [up to 3 months: *b* = −0.77, SE*b* = 0.16, *t*(68.86) = −4.66, *p* < 0.001, more than 3 months: *b* = −0.15, SE*b* = 0.17, *t*(80.57) < −1, *p* = 0.37], emotional well-being [up to 3 months: *b* = −0.62, SE*b* = 0.10, *t*(67.33) = −5.94, *p* < 0.001, more than 3 months: *b* = −0.27, SE*b* = 0.12, *t*(80.48) < −2.27, *p* < 0.05], loneliness [up to 3 months: *b* = 0.42, SE*b* = 0.12, *t*(67.36) = 3.33, *p* < 0.01, more than 3 months: *b* = 0.10, SE*b* = 0.11, *t*(79.03) < 1, *p* = 0.35], and happiness with the move [up to 3 months: *b* = −0.65, SE*b* = 0.24, *t*(71.99) = −2.68, *p* < 0.01, more than 3 months: *b* = 0.33, SE*b* = 0.20, *t*(80.67) = 1.68, *p* = 0.10]. The same pattern was found for the difference between the two groups in the effect of social approach motives on loneliness [up to 3 months: *b* = −0.63, SE*b* = 0.13, *t*(77.38) = −4.98, *p* < 0.001, more than 3 months: *b* = −0.27, SE*b* = 0.12, *t*(79.51) < −2.22, *p* < 0.05]. No other effects of social approach motives on subjective well-being were moderated by time since move.

To summarize the results of the two studies in terms of our hypotheses:

1. Outcomes of social approach and avoidance motives:
  1a. Results supported our expectations that social approach motives are positively associated with the quantity and the quality of social contacts. In line with hypotheses, social approach motives were negatively related to loneliness but, contrary to expectations, unrelated to physical and affective well-being.
  1b. Again supporting hypotheses, social avoidance motives were unrelated to the quantity of social contacts but negatively to their quality and to physical and emotional well-being.
2. Age-related differences:
  2a. Expectations regarding the quantity and quality of social interactions were supported: older adults reported fewer social contacts in general and negative interactions in particular when compared to younger adults.
  2b. There were no differences between younger and older adults concerning the association between social motives and their outcomes. This supports the stability hypothesis.
3. Time during the transition:
  In line with our hypothesis, the impact of social approach and avoidance motives decreased over the course of the transition phase. However, the decrease was more pronounced for the avoidance than for the approach motives and for subjective well-being than for behavior and cognitions.

## Discussion

Throughout life, we undergo transitions that position us in new social environments. Arguably one of the central factors contributing to the successful mastery of transitions is how well we can establish new positive social relationships. Hence, the current studies focused on the role of social approach and avoidance motives for the daily experience of and behavior during important developmental transitions in young and old adulthood, namely to move out of the parental home in young adulthood and to move into senior housing in old age. The central finding of the current studies is that social motives have a stable impact on subjective indicators of mastering a developmental transition across adulthood. In other words, the person-related variables continue to exert an important influence on daily behaviors and social experiences as well as indicators of subjective well-being during profound developmental transitions well into old age.

### The role of social motives for social transitions

Social approach motives were associated with frequent social interactions and actively approaching relationships. Additionally, in line with the exposure hypothesis by Gable (2006), the higher the social approach motives were, the more positive social experiences participants reported. One unexpected result was that social approach motives did not predict the frequency and active pursuit of interactions with persons to whom participants undergoing the transition felt less close or not close at all. This is surprising given that approach motives were related to entering more new social relationships. A possible interpretation of this result is that approach motivation makes people more desirable social partners such that others are more inclined to pursue them (Mehrabian, 1994) and making it unnecessary for highly approach motivated persons to seek out new social contacts with people to whom they do not feel close. An alternative explanation is methodological in nature. Interactions were defined as lasting at least 10 min (in order to avoid reporting biases). One possible consequence of this restriction is that all brief encounters with strangers involving “small talk” were not captured in the frequency measure. However, when participants were asked if they entered new social relationships on a given day, they might have nevertheless counted such short interactions.

Although social avoidance motives did not predict the frequency of (own and others’) negative behaviors, persons with high social avoidance motives felt more often rejected and less often accepted than persons with low social avoidance motives. At the same time, they reported lower subjective well-being. Apart from negative social cognitions, there were no behaviors or experiences associated with social avoidance motives. It seems that the social stress that avoidance-motivated persons experience is mostly expressed on the cognitive level and is less pronounced on the level of behavior. This result has potential implications for interventions geared at increasing the mastery of developmental transitions. Such interventions should focus on cognitions rather than behaviors associated with high avoidance motivation.

### The role of age for social transitions

Replicating previous findings (Lang and Carstensen, 1994; Carstensen et al., 1999; Lang, 2004), the frequency of interactions with close persons differed only marginally between young and older adults. Moreover, older adults were not lonelier or less satisfied with their daily social experience than younger adults but reported higher emotional well-being and satisfaction with the move. This replication of the findings concerning indicators of subjective and social well-being is noteworthy as the move into senior housing is often caused by health problems or the death of the partner (Pitts et al., 2005). Regarding the quality of social encounters, older adults reported less negative (own and others’) behavior and less negative cognitions about their social relationships. These results are in line with the notion that older adults avoid negative social experiences and that this strategy might be particularly adaptive in older adulthood (Charles et al., 2009).

To our knowledge, the present studies are the first to test age-related differences in the impact of social approach and avoidance motivation on subjective indicators of successfully mastering a profound developmental transition. However, the results contribute to the growing evidence for stability in the impact of motives on social cognition. Nikitin and Freund (2011) found no age-related differences regarding the predictive power of social motives on gaze times for angry and happy faces. Different to the controlled experimental setting of the gaze study, the current studies use a naturalistic setting and social cognitions and behaviors in everyday life. The results of the current studies attest to the important role of motivational dispositions for navigating developmental challenges and demands throughout adulthood.

### The role of time for social transitions

Taking a more narrow time frame and focusing on the duration of the transition, results of the current studies (see Results Section “Time Since Move as a Moderator of the Association Between Social Motives and Daily Outcomes”) show that the role of social motives for mastering the transitions wanes over time. This difference was much more pronounced for social avoidance than for social approach motives. One interpretation of the differential time course of the impact of social approach and avoidance motives could be related to our findings that social approach motives affect the active pursuit of positive daily social encounters, whereas social avoidance motives are unrelated to active socializing. Thus, social approach motivation is associated with actively construing and designing one’s social environment and creating environments that correspond to one’s approach motivation. Being in a transition phase, people who are highly socially approach motivated might rapidly begin to build for themselves a positive social environment and maintain this environment over time. This might lead to a continued influence of social approach motivation on social cognitions and behaviors as well as subjective well-being throughout the transition. In contrast, the impact of social avoidance motives might change with the occurrence of stressful experiences. Stressful experiences might increase during the transition because there are many new and unpredictable situations including potential misunderstandings, conflicts, and social rejection. Previous research (Gable, 2006) and findings from the current studies have shown that social avoidance motives are associated with stronger reactions to such negative situations. The longer the time from the transition, the fewer unpredictable and ambiguous situations appear. This might be the reason why, with increasing time from the transition, the effects of social avoidance motives decrease.

In fact, we even found a marginal positive association between social avoidance motives and satisfaction with the move after 3 months. More studies are needed to test if this result can be replicated and represents a substantive difference in the relationship between avoidance motives and different outcomes variables. An explanation of this finding could be that social avoidance motives lead to an expectation that one will not succeed in mastering the transition. At the beginning of the transition, which is the more difficult time due to the lack of familiarity with the new situation, this might be more likely to be true than not: people might have problems to socialize, to make new acquaintances, and, more generally, to feel in command of the new situation. Gradually, the new social situation becomes more predictable and less ambivalent, and by the mere passage of time, people also have more opportunities to meet and get to know other people (Asendorpf and Wilpers, 1998). Contrasting this experience with the negative expectations at the beginning might result in relief or even pride that, after all, one managed successfully an important social transition. This might be more strongly expressed in the evaluation of the situation (such as satisfaction with the move) than be reflected on the more global level of positive and negative emotions.

### Personality stability and change

The present studies contribute to the research on personality stability and change over the life span. Some researchers (e.g., Caspi et al., 2005) called for more data on the expression of personality through behavior in context. As Funder (2001) put it: “The empirical study of personality properly encompasses three elements: the person, the situation, and behavior (because) knowledge about any two of these should lead to an understanding about the third” (p. 210). Following these suggestions, the present studies investigated the relationship between person variables (social approach and avoidance motives; age), situation variables (time since a social transition), and social behavior and experience. With this, the current studies have shown that the situation-motive relation varies for social approach and avoidance motives, respectively. More specifically, social approach motives are more stable than social avoidance motives over the time of the transition. Further, the motive-behavior/experience relation also varied for social approach and avoidance motives. Social approach motives were associated more strongly with social behavior and avoidance motives with the social experience. These results contribute to the explanation of the robust finding that some people are more prone to change than others (Roberts et al., 2001). Our results are in line with the explanation by Roberts et al. (2001) that people who are interpersonally active, planful, and decisive (like those with strong social approach motivation) are less likely to change, probably because they exert more influence on their environment than others. Those who react rather than act (like those with strong social avoidance motivation) might be more influenced by their environment.

Finally, results of the present studies support recent findings that intra-individual stability in traits or dispositions increases from adolescence to young adulthood and then plateaus (Terracciano et al., 2010). According to the current studies, the dispositions of social approach and avoidance motives show context-related differential instability but age-related stability in adulthood.

### Limitations

One of the shortcomings of the present set of studies is that the time since moving into a new environment was longer for the sample of older than that of younger adults. We addressed this problem by controlling for time from move (or age, respectively) in the multilevel models. A logistically more challenging way of addressing this problem would be to start data-collection before the onset of the transition and follow individuals for exactly the same period of time before and during the transition. On a practical level, this would be very difficult to do for the sample of older adults because only a relatively small number of older adults move into senior housing in any given month.

Another limitation of the current studies is that subjective physical well-being was assessed by a single item. Moreover, this item might have produced biased answers as it was weighted in a positive direction (“Did you feel physically well?”). This would be a minor issue if Study 2 did not focus on older adults who have transitioned into a care facility and it is not unlikely that health issues were a reason for the move. Although a *t*-test of the difference between young and older adults in subjective physical well-being (aggregated over 14 days) revealed that older adults reported even better physical well-being (*M* = 4.12, SD = 1.17) than young adults (*M* = 3.60, SD = 1.17), *t*(154) = 2.72, *p* = 0.006, this does not mean that older adults were healthier than young adults. In fact, problems due to bad health might provide an alternative explanation of the reduced quantity of social encounters in the older group as compared to the younger group. Although there is some empirical evidence that the size of personal network cannot be explained by physical health alone (Lang and Carstensen, 2002), we cannot rule out that physical health might play a role for the social-network size in the present studies. Future studies might therefore directly assess physical complaints and test their role for social behavior beyond social motivation.

Another issue concerns possible different meanings of the two transitions. Although we tried to find two transitions that are similar in many ways, the transition from the parental home to a shared apartment might be associated with growth and maturation, whereas the transition to senior housing might be associated with loss and decline. Moreover, moving out from the parental home might represent a normative transition, whereas moving in senior housing might be more strongly associated with personal strains (loss of a partner, physical decline). Given this difference, the finding that older adults adjusted better to the transition might reflect older adults’ abilities to master challenges of later adulthood associated with decline and loss (e.g., Brandtstädter and Greve, 1994).

Another limitation of the current studies is that they did not include a group of middle-aged adults in the study. The reasons for this was that we tried to hold the transition as constant as possible for the different age groups. This was possible for the transition of moving into a new environment in young adults who start university and for older adults moving into senior housing. No such transition exists for middle-aged adults. Although middle-aged adults also move, they typically do not move to a different kind of housing arrangement. Nevertheless, the design of our studies leaves open whether the development of the association between the dispositions and their outcomes might be characterized by a curvilinear trend.

## Conclusion

Taken together, the present studies show that age and social motives independently affect daily social experiences and behaviors in a developmental transition. The positive effects of social approach motives were expressed by reports of frequent positive social contacts, whereas the positive effects of age were expressed by less frequent negative social contacts. Age did not moderate the association between social motives and outcomes of social functioning and successfully mastering the transition. Time during the transition, however, was an important moderator, particularly of the negative association between social avoidance motives and subjective well-being. It seems, then, that in some way time “heals” the negative consequences of social avoidance motives during a developmental transition, while the positive effects of approach motives are maintained over time.

## Author Note

This study was supported by Grant 100014_126868/1 (Project “Social Approach and Avoidance Motives – The Role of Age”) from the Swiss National Science Foundation (PIs: Jana Nikitin and Alexandra M. Freund). We are grateful to the Life-Management Lab for fruitful discussions of the work. We wish to thank Annina Singer and Christian Gross for their help with data collection. Moreover, we appreciate the time and effort the study participants invested in this research.

## Conflict of Interest Statement

The authors declare that the research was conducted in the absence of any commercial or financial relationships that could be construed as a potential conflict of interest.
